# Intravenous immunoglobulin for maintenance treatment of multifocal motor neuropathy: A multi‐center, open‐label, 52‐week phase 3 trial

**DOI:** 10.1111/jns.12268

**Published:** 2018-04-24

**Authors:** Satoshi Kuwabara, Sonoko Misawa, Masahiro Mori, Yuta Iwai, Kazuhide Ochi, Hidekazu Suzuki, Hiroyuki Nodera, Akira Tamaoka, Masahiro Iijima, Tatsushi Toda, Hiroo Yoshikawa, Takashi Kanda, Ko Sakamoto, Susumu Kusunoki, Gen Sobue, Ryuji Kaji, on behalf of the Glovenin‐I MMN Study Group, Satoshi Kuwabara, Masahiro Mori, Sonoko Misawa, Yuta Iwai, Kazuhide Ochi, Susumu Kusunoki, Hidekazu Suzuki, Ryuji Kaji, Hiroyuki Nodera, N. Matsui, Akira Tamaoka, A. Ishi, Gen Sobue, Masahiro Iijima, Tatsushi Toda, K. Sekiguchi, Hiroo Yoshikawa, A. Yamamoto, Takashi Kanda, T. Maeda, M. Tahara, M. Nakagawa, T. Mizuno

**Affiliations:** ^1^ Department of Neurology Chiba University Hospital Chiba Japan; ^2^ Department of Clinical Neuroscience and Therapeutics Hiroshima University School of Medicine Hiroshima Japan; ^3^ Department of Neurology Kindai University Faculty of Medicine Osaka Japan; ^4^ Department of Neurology Tokushima University School of Medicine Tokushima Japan; ^5^ Department of Neurology, Faculty of Medicine University of Tsukuba Ibaraki Japan; ^6^ Department of Neurology Nagoya University School of Medicine Aichi Japan; ^7^ Division of Neurology Kobe University Graduate School of Medicine Hyogo Japan; ^8^ Division of Neurology, Department of Internal Medicine Hyogo College of Medicine Hyogo Japan; ^9^ Department of Neurology Yamaguchi University Graduate School of Medicine Yamaguchi Japan; ^10^ Nihon Pharmaceutical Co.,Ltd Tokyo Japan; ^11^ Research Division of Dementia and Neurodegenerative Disease Nagoya University Graduate School of Medicine Aichi Japan; ^12^ Department of Neurology Chiba University Hospital Chiba Japan; ^13^ Department of Clinical Neuroscience and Therapeutics Hiroshima University School of Medicine Hiroshima Japan; ^14^ Department of Neurology Kindai University Faculty of Medicine Osaka Japan; ^15^ Department of Neurology Tokushima University School of Medicine Tokushima Japan; ^16^ Department of Neurology, Faculty of Medicine University of Tsukuba Ibaraki Japan; ^17^ Department of Neurology Nagoya University School of Medicine Aichi Japan; ^18^ Research Division of Dementia and Neurodegenerative Disease, Nagoya University Graduate School of Medicine Aichi Japan; ^19^ Department of Neurology Nagoya University School of Medicine Aichi Japan; ^20^ Division of Neurology Kobe University Graduate School of Medicine Hyogo Japan; ^21^ Present Address: Department of Neurology The University of Tokyo Tokyo Japan; ^22^ Division of Neurology Kobe University Graduate School of Medicine Hyogo Japan; ^23^ Division of Neurology, Department of Internal Medicine, Hyogo College of Medicine Hyogo Japan; ^24^ Department of Neurology Yamaguchi University Graduate School of Medicine Yamaguchi Japan; ^25^ Department of Neurology, Utano National Hospital Kyoto Japan; ^26^ Department of Neurology Kyoto Prefectural University of Medicine Kyoto Japan

**Keywords:** clinical trial, efficacy, intravenous immunoglobulin, multifocal motor neuropathy, safety

## Abstract

Intravenous immunoglobulin (IVIg) therapy is currently the only established treatment in patients with multifocal motor neuropathy (MMN), and many patients have an IVIg‐dependent fluctuation. We aimed to investigate the efficacy and safety of every 3 week IVIg (1.0 g/kg) for 52 weeks. This study was an open‐label phase 3 clinical trial, enrolling 13 MMN patients. After an induction IVIg therapy (0.4 g/kg/d for 5 consecutive days), maintenance dose (1.0 g/kg) was given every 3 weeks for 52 weeks. The major outcome measures were the Medical Research Council (MRC) sum score and hand‐grip strength at week 52. This trial is registered with http://clinicaltrials.gov, number NCT01827072. At week 52, 11 of the 13 patients completed the study, and all 11 had a sustained improvement. The mean (SD) MRC sum score was 85.6 (8.7) at the baseline, and 90.6 (12.8) at week 52. The mean grip strength was 39.2 (30.0) kPa at the baseline and 45.2 (32.8) kPa at week 52. Two patients dropped out because of adverse event (dysphagia) and decision of an investigator, respectively. Three patients developed coronary spasm, dysphagia, or inguinal herniation, reported as the serious adverse events, but considered not related with the study drug. The other adverse effects were mild and resolved by the end of the study period. Our results show that maintenance treatment with 1.0 g/kg IVIg every 3 week is safe and efficacious for MMN patients up to 52 weeks. Further studies are required to investigate optimal dose and duration of maintenance IVIg for MMN.

## 1 INTRODUCTION

Multifocal motor neuropathy (MMN) is an immune‐mediated peripheral neuropathy, characterized by progressive asymmetric distal muscle weakness predominantly in the upper extremities, and pure motor involvement.^1^, ^2^ Current therapeutic options for MMN are limited, and intravenous immunoglobulin (IVIg) therapy is only one established treatment; as MMN patients do not respond to corticosteroids or plasma exchange.^3^, ^4^

So far, 5 randomized, placebo‐controlled trials of IVIg for MMN have shown significant short‐term improvement in muscle strength, up to 12 weeks.^5^, ^6^, ^7^, ^8^, ^9^ Based on these data, the joint European Federation of Neurological Societies/Peripheral Nerve Society (EFNS/PNS) taskforce recommended IVIg (2 g/kg) as a first‐line treatment for MMN.^1^ The taskforce also proposed that “if this induction therapy is effective, repeated IVIg should be considered with the frequency of maintenance therapy dose are 1 g/kg every 2‐4 weeks, or 2 g/kg every 1‐2 months”. However, there is limited evidence for the dose and interval of maintenance IVIg.

No long‐term clinical trials that investigated the efficacy and safety of IVIg in MMN have been performed. Four retrospective studies have described MMN patients who have received repeated IVIg therapy over several years, and suggested that it may be used as the long‐term treatment option for MMN.^10^, ^11^, ^12^, ^13^ In these reports, the IVIg regimen, interval, and treatment response varied among patients; some experienced sustained remission and others had gradual progression of muscle weakness presumably because of Wallerian degeneration. The aim of this clinical trial is to study the efficacy and safety of maintenance IVIg (1.0 g/kg every 3 weeks) for 52 weeks in patients with MMN.

## 2 MATERIALS AND METHODS

### 2.1 Study design and patients

This study was a multi‐center, single‐arm open phase 3 trial conducted at 11 Japanese tertiary hospitals. The study procedures were in accordance with the Declaration of Helsinki, and Japanese Good Clinical Practice criteria, and approved by the internal review board of each hospital. This study is registered with http://clinicaltrials.gov, number NCT01827072.

A total of 13 patients with definite or probable MMN according to the EFNS/PNS clinical diagnostic criteria were enrolled.^4^ The inclusion criteria were defined as (1) requiring a high‐dose IVIg treatment (having declining motor function), (2) no additional immunotherapy, or if already treated, not increasing dose of agents for MMN from 30 days prior to consent, (3) at week 4 in the induction period, Medical Research Council (MRC) sum score^14^ in 2 or more muscles improved by 1 point or more, compared to that on week 1 (before treatment) in the induction period, (4) age 20 years or older. The main exclusion criteria were receiving (1) plasma exchange within 3 months prior to consent, (2) natalizumab or rituximab within 6 months prior to consent, (3) interferon beta within 6 months prior to consent, (4) high‐dose IVIg (1.0 g/kg or more) within 8 weeks prior to consent, and (5) any IVIg treatment within 3 weeks prior to consent.

### 2.2 Procedures

**Figure 1:**
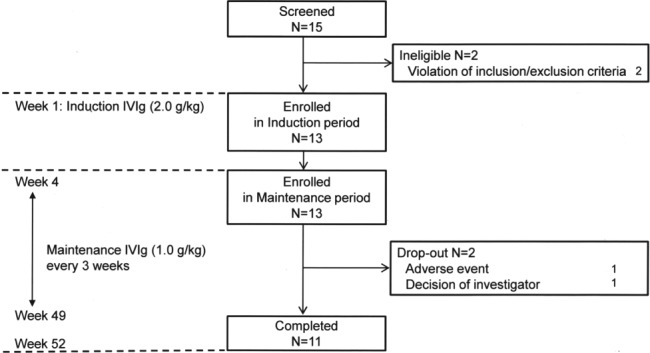
Study design and trial profile

The study design and trial profile are shown in Figure 1. After screening, IVIg (0.4 g/kg/d for consecutive 5 days) was administered as the induction treatment. This visit was regarded as week 1 in the induction period. After 3 weeks, IVIg was then administered (1.0 g/kg/d for 1 day, or 0.5 g/kg/d for consecutive 2 days) as the maintenance treatment. This visit was regarded as week 4 in the study. Glovenin‐I (freeze‐dried polyethylene glycol‐treated human immunoglobulin, Nihon Pharmaceutical Co., Ltd., Tokyo, Japan) was used for maintenance treatment. The maintenance IVIg was administrated every 3 weeks from week 4 to week 49 in the maintenance period, with observation conducted until week 52. If additional treatment for MMN was needed, the patient dropped out.

For neurological assessment, MRC sum score, hand‐grip strength, and Guy's Neurological Disability Scale (GNDS) score^15^ were assessed at each visit. Each neurological assessment, except at week 52, was conducted before administration of IVIg.

### 2.3 Outcome measures

The outcome measures were MRC sum score, hand‐grip strength score, and GNDS score. Difference in the mean scores at week 1 and week 52 was calculated. These measures were not distinguished as primary and secondary because the number of MMN patients included in this trial was small. Safety assessments included any adverse event during the study period.

### 2.4 Data analyses

Analyses were performed by the full analysis set which included all patients who received the drug at least once. Missing data of MRC sum score, hand‐grip strength, and GNDS score were input by data at the last visit (the last observation carried forward method). Confidence interval (CI) was calculated using the Clopper‐Pearson exact method for both periods. The target number of 10 patients was set in view of the rarity of MMN. All analyses were performed with the statistical software package SAS, release 9.2 (SAS Institute Inc., Cary, North Carolina). Statistical comparison was not performed because of the small number of patients included.

## 3 RESULTS

### 3.1 Patient disposition

The study was conducted from June 2013, and ended in June 2015. A total of 15 patients were screened. Two patients were ineligible, and the remaining 13 were enrolled (Figure 1); 12 of them were responders to prior IVIg treatment (2.0 g/kg), and the remaining 1 was treatment‐naïve. During the study, 2 patients discontinued the study because of an adverse event (dysphagia) and decision of the investigator (slight decline of muscle strength), respectively. Of the 13 patients, 11 completed the 52‐week study.

Table 1 shows patients' baseline characteristics. Eight patients had definite MMN and 5 had probable MMN. The mean age was 60 years (range, 44‐77 years) and the mean disease duration was 54 months (range, 7‐205 months); 92% of the patients had received IVIg treatment before the study. The mean baseline serum IgG level was 1296 mg/dL (normal range, 900‐1800 mg/dL).

**Table 1:** Demographics and baseline disease characteristics

		Category	All patients (*n* = 13)
Gender (%)		Man	10 (76.9)
Age (y)		<65 (%)	8 (61.5)
		≥65 (%)	5 (38.5)
		Mean (SD)	60.3 (10.9)
		Range	44‐77
Duration of MMN (mo)		Mean (SD)	54.1 (54.8)
		Range	7‐205
Number of relapse over the 3 years prior to consent		Mean (SD)	6.9 (6.2)
		Range	1‐20
MMN treatment history (%)		IVIg	12 (92.3)
		Plasma exchange	1 (7.7)
		Others	3 (23.1)
MMN diagnostic type		Definite (%)	8 (61.5)
		Probable (%)	5 (38.5)
MRC sum score on week 1		Mean (SD)	85.6 (8.7)
		Range	72‐98
MRC sum score on week 4		Mean (SD)	90.5 (9.3)
		Range	73‐100
Hand‐grip strength (kPa) on week 1	Dominant	Mean (SD)	39.2 (30.0)
		Range	8‐97
	Non‐dominant	Mean (SD)	29.8 (22.7)
		Range	0‐74
Hand‐grip strength (kPa) on week 4	Dominant	Mean (SD)	49.6 (31.8)
		Range	10‐110
	Non‐dominant	Mean (SD)	39.5 (26.5)
		Range	0‐81
GNDS sum score on week 1		Mean (SD)	3.4 (1.4)
		Range	2‐6
GNDS sum score on week 4		Mean (SD)	2.6 (1.3)
		Range	1‐5
Previous medical history		No	7 (53.8)
		Yes	6 (46.2)
Serum IgG concentration (mg/dL) on week 1		Mean (SD)	1296 (321.8)
		Range	888‐1955

GNDS, Guy's Neurological Disability Scale; IVIg, intravenous immunoglobulin; MMN, multifocal motor neuropathy; MRC, Medical Research Council.

### 3.2 Efficacy

**Figure 2:**
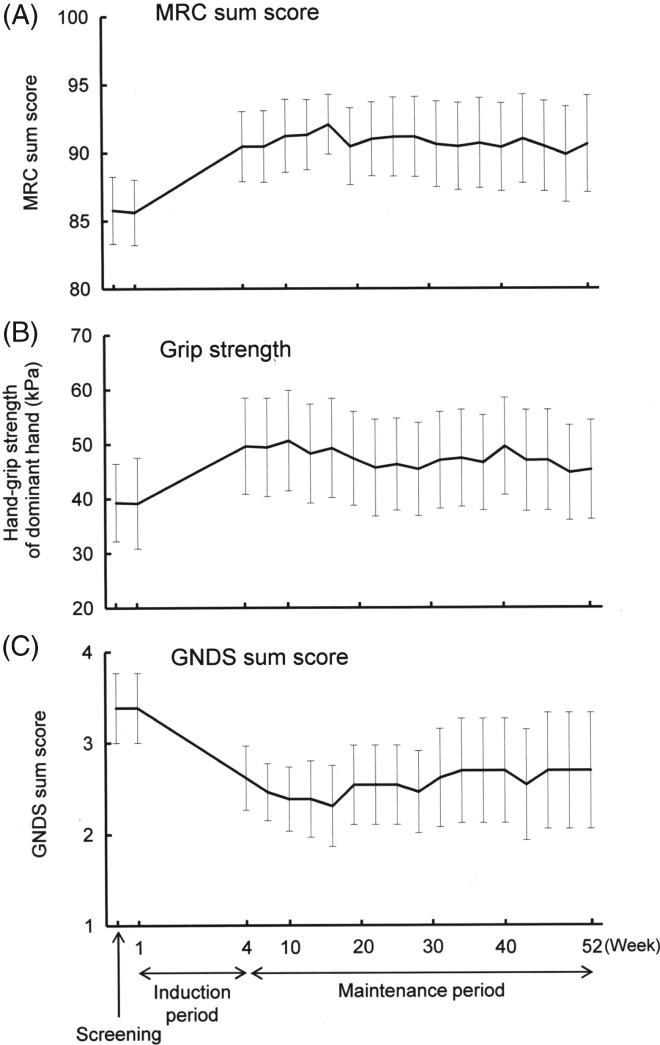
Transition diagram of symptoms in multifocal motor neuropathy (MMN). (A) Medical Research Council (MRC) sum score, (B) hand‐grip strength of dominant hand, (C) Guy's Neurological Disability Scale (GNDS) sum score. The visit interval was every 3 weeks in maintenance period. Error bars represent SEM

Results of the outcome measures are shown in Table 2. The mean value of MRC sum score, hand‐grip strength, and GNDS score improved from the baseline (week 1) to week 52, except GNDS lower limb score. The sequential changes in the MRC sum score, hand‐grip strength, and GNDS score are shown in Figure 2. All measures improved after induction therapy and the improvement continued through week 52 in 11 of the 13 patients. Whereas some fluctuation of the measures occurred during maintenance IVIg treatment, the 11 patients finally had sustained improvement at week 52, the measure being similar to those at the baseline (week 4). Serum IgG levels were higher at week 4 than those at week 1, and maintained at approximately 2000 mg/dL up to week 52.

**Table 2:** Efficacy of IVIg in patients with MMN

	Induction period (*n* = 13) Week 1	Maintenance period (*n* = 13)	
		Week 4	Week 52
MRC sum score	85.6 (8.7)	90.5 (9.3)	90.6 (12.8)
Hand‐grip strength (kPa)			
Dominant hand	39.2 (30.0)	49.6 (31.8)	45.2 (32.8)
Non‐dominant hand	29.8 (22.7)	39.5 (26.5)	41.1 (28.6)
GNDS score			
Upper limb	2.8 (0.8)	2.2 (1.2)	1.8 (1.4)
Lower limb	0.6 (1.0)	0.5 (0.7)	0.8 (1.1)
Sum score	3.4 (1.4)	2.6 (1.3)	2.7 (2.3)
Serum IgG (mg/dL)	1296 (322)	2070 (341)	1974 (363)^a^

Data are shown as mean (SD). GNDS, Guy's Neurological Disability Scale; IVIg, intravenous immunoglobulin; MMN, multifocal motor neuropathy; MRC, Medical Research Council.

a *n* = 11.

### 3.3 Safety

A total of 12 (92.3%) of the 13 patients experienced adverse events (95% CI: 64.0%‐99.8%). Table 3 shows details of adverse events with the incidence of 15% or more. Frequent events were nasopharyngitis (38.5%), headache (23.1%), and contusion (23.1%). Additionally, adverse drug reactions were observed in 69.2% (9/13 patients, 95% CI, 38.6%‐90.9%). No death occurred during the study.

**Table 3:** Adverse events reported in ≥15% of patients

Total patients	*N* = 13	
Patients developing adverse events	*N* = 12	
Rate of developing adverse events	92.3%	
Total number of developing adverse events	*N* = 79	
**Adverse event name (PT)**	**Number of patients**	**(%)**
Nasopharyngitis	5	38.5
Headache	3	23.1
Contusion	3	23.1
Epistaxis	2	15.4
Dental caries	2	15.4
Diarrhea	2	15.4
Dysphagia	2	15.4
Rash	2	15.4

Medical dictionary for Regulatory Activities (MedDRA), version 18.0.

Three patients experienced serious adverse events, including coronary artery stenosis (*n* = 1), dysphagia (*n* = 1), and inguinal hernia (*n* = 1). None of them was considered to relate with IVIg.

## 4 DISCUSSION

The present study showed that after conventional induction IVIg therapy, maintenance IVIg treatment (1.0 g/kg) every 3 week resulted in sustained clinical improvement for 52 weeks. The results were supported by sequential findings of MRC sum score, grip strength, and GNDS score. The maintenance IVIg therapy was not associated with clinically significant adverse effects. Our results show the long‐term efficacy and safety of the maintenance IVIg.

In previous retrospective studies, a variable maintenance IVIg regimens, such as 1.0 g/kg every 2 to 4 weeks, or 2 g/kg every 1 to 2 months, were used dependent on patients' condition. Whereas disease activity, immunoglobulin metabolism, and response to treatment are presumably different among patients with MMN, the optimal dose and interval may be determined according to patients' situation. Nevertheless, this study revealed uniform regular maintenance IVIg administration was successful in almost of the MMN patients enrolled. The regimen used in this trial can be an option to achieve sustained remission in MMN at least for 52 weeks.

In patients with chronic inflammatory demyelinating polyneuropathy (CIDP), approximately 25% of patients have long‐lasting remission without immunological treatment,^16^, ^17^ and prolonged maintenance therapy could be over treatment. However, such remitting course is rare for MMN,^10^ and MMN patients may require maintenance therapy for more than 52 weeks. Therefore the duration of IVIg therapy, as well as, the optimal regimen, for longer maintenance IVIg for MMN patients should be evaluated in future studies.

A recent clinical trial for CIDP using the same maintenance dose (1.0 g/kg) and interval (every 3 weeks) for 52 weeks has also shown similar long‐term efficacy.^18^ In that study, 2 of the 49 enrolled elderly patients with hypertension or diabetes developed cerebral infarction. Whereas thromboembolic events did not occur in the present study, hyperviscosity‐induced thrombotic complications should be carefully monitored during a long‐term IVIg treatment.

In conclusion, 52‐week maintenance IVIg therapy appears to be safe and efficacious to prevent a relapse for MMN patients. The longer‐term safety and efficacy, and need of dose adjustment should be investigated in future studies.

## Glovenin‐I MMN Study Group:

S.K., M.M., S.M., Y.I. (Department of Neurology, Chiba University Hospital, Chiba, Japan); K.O. (Department of Clinical Neuroscience and Therapeutics, Hiroshima University School of Medicine, Hiroshima, Japan); S.K., H.S. (Department of Neurology, Kindai University Faculty of Medicine, Osaka, Japan); R.K., H.N., N. Matsui (Department of Neurology, Tokushima University School of Medicine, Tokushima, Japan); A.T., A. Ishi (Department of Neurology, Faculty of Medicine, University of Tsukuba, Ibaraki, Japan); G.S. (Department of Neurology, Nagoya University School of Medicine, Aichi, Japan, Research Division of Dementia and Neurodegenerative Disease, Nagoya University Graduate School of Medicine, Aichi, Japan), M.I. (Department of Neurology, Nagoya University School of Medicine, Aichi, Japan); T.T. (Division of Neurology, Kobe University Graduate School of Medicine, Hyogo, Japan, Present Address: Department of Neurology, The University of Tokyo, Tokyo, Japan), K. Sekiguchi (Division of Neurology, Kobe University Graduate School of Medicine, Hyogo, Japan); H.Y., A. Yamamoto (Division of Neurology, Department of Internal Medicine, Hyogo College of Medicine, Hyogo, Japan); T.K., T. Maeda (Department of Neurology, Yamaguchi University Graduate School of Medicine, Yamaguchi, Japan); M. Tahara (Department of Neurology, Utano National Hospital, Kyoto, Japan); M. Nakagawa, T. Mizuno (Department of Neurology, Kyoto Prefectural University of Medicine, Kyoto, Japan).
